# Systematic Development of a Simultaneous Determination of Plastic Particle Identity and Adsorbed Organic Compounds by Thermodesorption–Pyrolysis GC/MS (TD-Pyr-GC/MS)

**DOI:** 10.3390/molecules25214985

**Published:** 2020-10-28

**Authors:** Julia Reichel, Johanna Graßmann, Thomas Letzel, Jörg E. Drewes

**Affiliations:** 1 Chair of Urban Water Systems Engineering, Technical University of Munich, Am Coulombwall 3, 85748 Garching, Germany; julia.reichel@tum.de (J.R.); j.grassmann@tum.de (J.G.); t.letzel@tum.de (T.L.); 2 AFIN-TS GmbH, Am Mittleren Moos 48, 86167 Augsburg, Germany

**Keywords:** microplastic, nanoplastic, thermodesorption, pyrolysis, desorption

## Abstract

Micro-, submicro- and nanoplastic particles are increasingly regarded as vectors for trace organic chemicals. In order to determine adsorbed trace organic chemicals on polymers, it has usually been necessary to carry out complex extraction steps. With the help of a newly designed thermal desorption pyrolysis gas chromatography mass spectrometry (TD-Pyr-GC/MS) method, it is possible to identify adsorbed trace organic chemicals on micro-, submicro- and nanoparticles as well as the particle short chain polymers in one analytical setup without any transfers. This ensures a high sample throughput for the qualitative analysis of trace substances and polymer type. Since the measuring time per sample is only 2 h, a high sample throughput is possible. It is one of the few analytical methods which can be used also for the investigation of nanoplastic particles. Initially adsorbed substances are desorbed from the particle by thermal desorption (TD); subsequently, the polymer is fragmented by pyrolysis (PYR). Both particle treatment techniques are directly coupled with the same GC-MS system analyzing desorbed molecules and pyrolysis products, respectively. In this study, we developed a systematic and optimized method for this application. For method development, the trace organic chemicals phenanthrene, α-cypermethrin and triclosan were tested on reference polymers polystyrene (PS), polymethyl methacrylate (PMMA) and polyethylene (PE). Well-defined particle fractions were used, including polystyrene (sub)micro- (41 and 40 µm) and nanoparticles (78 nm) as well as 48-µm sized PE and PMMA particles, respectively. The sorption of phenanthrene (PMMA << PS 40 µm < 41 µm < PE < PS 78 nm) and α-cypermethrin (PS 41 µm < PS 40 µm < PE < PMMA < PS 78 nm) to the particles was strongly polymer-dependent. Triclosan adsorbed only on PE and on the nanoparticles of PS (PE < PS78).

## 1. Introduction

More than 300 million tons of plastics are annually produced worldwide and about 8 million tons migrate from land surfaces into the ocean [1,2]. It is estimated that currently more than five trillion plastic particles with a total weight of over 250,000 tons float in the oceans [3]. Most of them are microplastics with a size of less than 5 mm [4]. To date, no uniform definition has been established to distinguish between micro- and nanoplastics [5]. Plastic particles with a size between 1 mm and 1 µm are usually referred to as microplastics, while plastic particles smaller than 1 µm are defined as meso- or nanoplastic or submicroparticles [6,7]. Micro-, submicro- and nanoplastics may potentially not only be harmful by themselves but also serve as vectors due to ad- or absorbed contaminants [5]. Detailed and robust analysis of these sorbed contaminants is challenging and requires several analytical steps [5,8,9,10]. Using established analytical methods, such as thermal extraction desorption gas chromatography mass spectrometry (TED-GC/MS), double-shot analytical pyrolysis and sequential pyrolysis, it has already been shown that it is possible to detect additives and identify polymers with one analytical setup [11,12,13,14]. Considering established methods to analyze sorbed substances, initially, trace organic chemicals have to be extracted with solvents such as n-hexane, cyclohexane or dichloromethane [8,15,16] prior to analysis via GC/MS [10,15,17], LC/MS [8,9], or using radioisotopic marked trace organic chemicals [18,19]. Simultaneous identification of the polymer is not possible.

So far, several methods exist to identify the polymers. Currently, spectroscopic methods, such as Raman microscopy (RM) or Fourier transform infrared spectroscopy (FTIR), and thermoanalytical methods, such as pyrolysis gas chromatography mass spectrometry (Pyr-GC-MS) or thermal extraction desorption gas chromatography mass spectrometry (TED-GC-MS), are used [14,20,21,22,23,24]. Especially thermoanalytical methods are becoming more important for general identification and quantification of micro- and nanoplastics [25,26].

As the surface area of plastic particles increases the smaller the particle becomes, it is assumed that smaller particles are of higher ecotoxicological relevance since the capacity for adsorption of trace organic chemicals increases [4,27,28]. However, the effective surface area of nano-sized particles can be limited due to particle aggregation by increased hydrodynamic diameter [29]. Already, with other particle types (e.g., titanium particles or magnesium oxide particles), it could be shown that the relative sorption increases, the smaller the particle becomes [30,31]. These observations can be supported by research with (sub)micro- and nanoplastics, where sorption of polychlorinated biphenyls on nano-PS is 1–2 orders of magnitude higher than on micro-PE [16].

The aim of this study is to develop an innovative analytical method of combined thermodesorption- and pyrolysis-gas chromatography/mass spectrometry (TD-GC/MS + Pyr-GC/MS) in order to enable the identification of sorbed organic chemicals and the type of polymer in one single analytical setup. For doing so, initially, the trace organic chemicals are desorbed from the particles by thermodesorption and analyzed using GC/MS. Subsequently, the polymers are decomposed by pyrolysis and the decomposition products and by this the type of polymer and contained additives are identified via GC/MS analysis. The fields of application of such a method are mainly directed to environmental screening studies, e.g., those resulting from ecotoxicological assays employing micro- or nanoplastics and sorbed organic chemicals, where a quick analytical method is desired that is able to handle high sample numbers. This method is especially suitable for water samples. After filtration of the samples, the inorganics should be removed for analysis. For an analysis of the sorbed trace substances, the samples should also be as free as possible from organic substances, such as biofilms. A quantification of the trace organic chemicals sorbed on the particles is beyond the scope of this paper and will be performed in the future.

## 2. Analytical Systems, Materials and Methods

### 2.1. Instrumental Systems

**Figure 1:**
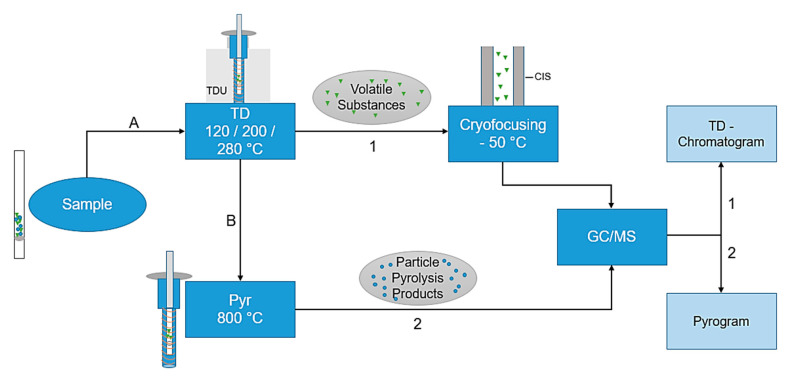
Flowchart of the thermodesorption- and pyrolysis-gas chromatography/mass spectrometry (TD-Pyr-GC/MS) analysis. First, (A) the sample is thermodesorbed (120–280 °C), thereby desorbing the volatile substances and cryofocusing them in the Cooled Injections System (CIS) at −50 °C. This is followed by a transfer to the GC column with an MS analysis (TD-GC/MS). The same sample (B) is subsequently pyrolyzed at 800 °C, followed by a GC/MS analysis (Pyr-GC/MS). The evaluations are carried out using the TD-Chromatogram and the Pyrogram.

**Figure 2:**
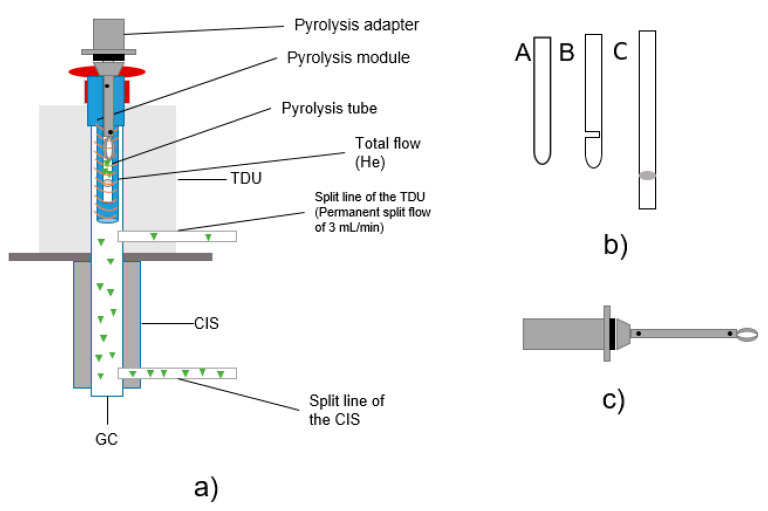
(**a**) Structure TD-GC/MS + Pyr-GC/MS; (**b**) design of different pyrolysis tubes (sample tubes); **c**) drawing of pyrolysis transport adapter.

The combined thermodesorption- and pyrolysis-gas chromatography/mass spectrometry (TD-GC/MS + Pyr-GC/MS) analysis was performed using a Gerstel Thermal-Desorption-Unit (TDU) 2 equipped with a TDU pyrolysis module, a Gerstel Multi-Purpose-Sampler (MPS) robotic^pro^, a Cooled Injections System (CIS) 4 with Controller C506 and an Agilent 7890B gas chromatograph equipped with an DB-5MS Ultra Inert column coupled with an electron ion source to an Agilent 5977B MSD mass spectrometer. The particle samples are directly transferred into the pyrolysis module by the MPS for further analysis. A preprogrammed temperature protocol is executed to evaporate the volatile substances in a first step. Subsequently, in the CIS, the substances are trapped and then totally transferred to the GC column entry by CIS heating. Due to the direct coupling of the pyrolysis module, it is possible to execute first a thermodesorption step (TD-GC/MS), subsequently followed by an independent pyrolysis step (Pyr-GC/MS). The total process is visualized in Figure 1; a more detailed scheme of the pyrolysis unit is shown in Figure 2a.

TD-GC/MS: The final thermal desorption temperature was set to three endpoints (120 °C, 200 °C and 280 °C). The initial temperature of the thermal desorption unit (TDU) is set to 20 °C with a delay of 0.3 min and an initial hold time of 1.0 min. The sample is heated to the respective endpoint temperature of 120 °C/200 °C/280 °C at 60 °C/min over a gradient and held for 5 min (Figure 1, step A). The desorption of the sample in the TDU occurs subsequently after the injection into the cooled injection system (CIS). In the CIS, the substances are trapped at −50 °C and heated to 120 °C/200 °C/280 °C with a 10 °C/min gradient, in order to transfer the trapped analytes onto the GC column (Figure 1, arrow 1). The transfer line temperature is 300 °C; the desorption mode is split-less, except for the permanent split flow. The GC/MS method (Figure 1; line 1 on the right) is adopted from Ochiai et al. (2005) [32]. However, the temperature for cryo-focusing is set to −50 °C instead of −150 °C. The initial temperature is set to 70 °C and is held for 2 min. The sample is then heated up to 150 °C with a gradient of 25 °C/min, followed by a heating rate of 3 °C/min to 200 °C. In the last step, the sample is heated by a gradient of 8 °C/min to 300 °C.

Pyr-GC/MS: Next, a pyrolysis step is performed (Figure 1, step B). The initial temperature of the TDU in the pyrolysis step is set to 50 °C with a hold time of 5.4 min. The sample is heated at 720 °C/min to 320 °C and this temperature is held for 1.4 min. After 0.3 min, the pyrolysis module heats the sample to a final temperature of 800 °C with a follow up time of 5 min. The transfer temperature is set to 350 °C and the desorption mode is split-less. The pyrolysis products are trapped in the CIS at 350 °C (Figure 1, arrow 2). The sample injection in the CIS is operated in split mode, i.e., the inlet ratio into the column is set to 100:1. The GC/MS method (Figure 1; line 2 on the right) is as follows: the initial temperature is set to 50 °C and is held for 2 min. The sample is then heated to 320 °C with a 10 °C/min gradient. This temperature is maintained for 3 min. Helium is used as carrier gas. In order to detect carry-over or impurities, an empty tube is measured after each sample.

The particle samples to be tested were placed in pyrolysis tubes for subsequent analysis (see Figure 2a). For the sample transfer into the CIS, three different pyrolysis tubes were tested. All were made of quartz glass and purchased from Gerstel GmbH (Mühlheim an der Ruhr, Germany). Since the design of the sample holder is an essential aspect regarding potential carry-over [33], several tube types were tested (visual setup details, see Figure 2b), including the following: pyrolysis tubes (A) with one open end and one closed end with a length of 17 mm, (B) with one open end and one closed end with an additional slot of 17 mm in length and (C) with two open ends and a length of 25 mm sealed with quartz wool. Thus, quartz wool is only used in type (C) and is contained in the tube and in contact with the sample. With the closed type (A) tube, the carrier gas flow is non-uniform; in the slotted type (B) tube, a more uniform carrier gas flow is ensured [33]. (C) is open at both ends, which should improve the carrier gas flow. The weight of the pyrolysis tubes was between 90 and 110 mg.

### 2.2. Materials and Methods

The reference particles were free of additives and provided by BS Partikel GmbH (Mainz, Germany). Polystyrene (PS) suspended in ethanol was supplied in the sizes of 78 nm and 41 µm, respectively. Further dry PS particles were delivered in sizes of 40 µm, and the polymethyl methacrylate (PMMA) and polyethylene (PE) particles in sizes of 48 µm, respectively. All PS particles were spherical and the shape of PMMA and PE particles was unknown. Phenanthrene (CAS: 85-01-8) and triclosan (CAS: 3380-34-5) were purchased from Sigma-Aldrich (Taufkirchen, Germany) and α-cypermethrin (CAS: 67375-30-8) from greyhoundchrom (Birkenhead, UK). All chemicals were stored at 4 °C. Methanol (≥99.8%) in HPLC-grade was purchased from VWR (Ismaning, Germany). Ultrapure water with pH 5 was obtained from a Sartorius arium pro ultrapure water system (Göttingen, Germany). For the weighing of the samples, a Sartorius Cubis ^®^ Ultramicro Balance (Göttingen, Germany) was used.

### 2.3. Sample Preparation for Reference and Ecotoxicological Samples

Initial experiments were conducted for TD temperature optimization (with lowest expected pyrolysis products) and no trace organic chemicals. The final sets of TD temperatures were observed at 120/200/280 °C to determine that no pyrolysis products occurred during the TD step.

**Figure 3:**
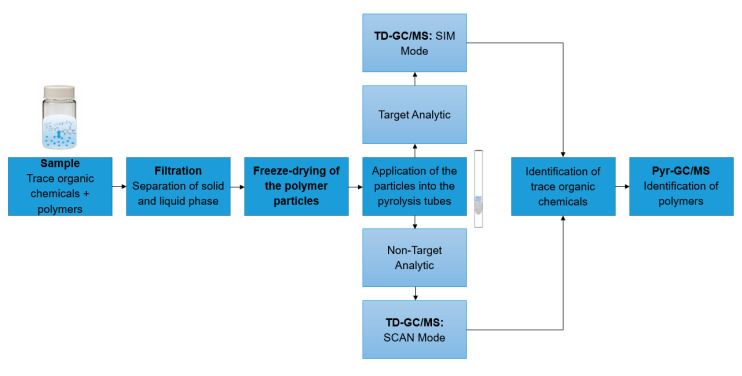
Workflow of sample analysis: First, the samples to be analyzed are filtered to separate the solid from the liquid phase. Then, the samples are applied into the pyrolysis tubes. Depending on the test setup, a non-target analytic (SCAN mode) or a target analytic (SIM mode) can be performed. Finally, a TD-Pyr-GC/MS analysis is conducted.

In all experiments, 10 mg of particles were suspended in 10 mL ultrapure water. The suspension was shaken for 1 h at room temperature at 1000 rpm. The solution was filtered by vacuum filtration to obtain dry particles. The particles were scraped off the filter with a spatula and directly transferred into a vial. The particles were freeze-dried for one hour. Subsequently, the particles were weighed into the pyrolysis tubes with a minimum and maximum weight of 30–80 to ensure reproducible results. The particles were transferred into the pyrolysis tubes using a syringe cannula. Each sample was weighed three times and the mean value was calculated to increase reproducibility. This sample tube was then subjected to TD-Pyr-GC/MS analysis. The entire sample preparation is summarized in the workflow in Figure 3. For the preparation of environmental samples from, for example, ecotoxicological assays, the sample preparation was carried out analogously.

In the sorption experiments with the target substances phenanthrene, α-cypermethrin and triclosan, the principle was the same as described above without trace organic chemicals. In these studies, the trace organic chemicals were added to the 10-mg particles in concentrations of 1000 µg/L and 100 µg/L. For the preparation of the stock solution of 1000 mg/L, the trace organic chemicals were dissolved in methanol and diluted further in methanol. The particles were suspended in 10 mL ultrapure water and treated as described above. The experiments for the measurements in SCAN and SIM mode were performed on PS 78 nm particles. The concentration was 1000 µg/L for each trace organic chemical. All experiments were carried out in triplicate on two different days, i.e., in total, six replicates.

### 2.4. Evaluation of the TD-Pyr-GC/MS Data

The mass spectrometer operated in full-scan mode (*m/z* range 40 to 550) with electron impact ionization (70 eV) for non-target analysis. For a target analysis, PS 78-nm particles and the trace organic chemicals phenanthrene (*m/z* 178), α-cypermethrin (*m/z* 163, 181, 165, 91, 77) and triclosan (*m/z* 290, 288, 218) were measured as references in SIM mode. Data analysis was conducted with Mass Hunter Workstation Software (Ver.B.08.000, Agilent). The identification of the substances was validated via MS spectra, and a NIST database comparison was conducted, as well as the retention index (RI) comparison [34].

Specific mass spectrometric signals were selected (Table 1 and Table 2) to extract and identify the data of the different polymers or trace organic chemicals (by means of their pyrolysis products). The data were standardized to make the sorption of trace organic chemicals on the particles comparable. The peak areas obtained after the specific extraction were divided by the weighed particle amount.

Three different polymers (PE, PS and PMMA) were selected for thermodesorption temperature optimization. Characteristic fragments were chosen for the unique identification of the individual polymers. Characteristic fragments that can be used for the identification of PS are the styrene mono-, di- and trimer. However, the monomer is also present in environmental samples because it also occurs in biogenic polymers, such as chitin or wool fibers [34], thus it can also be detected in the pyrolysis step. Therefore, solely the dimer and trimer serve as final indicator fragment for PS samples. The decomposition into small aliphatic chains (saturated, mono-saturated and di-unsaturated hydrocarbons) is typical for PE [22,35,36]. In environmental matrices, mono-unsaturated and saturated hydrocarbons also occur naturally since they result, for instance, from the decomposition of fatty acids and lipids [37]. Therefore, exclusively the di-unsaturated hydrocarbons (C13, C14 and C16) were used for the identification of PE [38]. The only pyrolysis product that could be detected reproducibly for PMMA was methyl methacrylate, which was used for identification.

The sorption data were evaluated with respect to sample ‘peak area’ to ‘particle weight’ ratio and the ratio of ‘peak area’ to ‘particle surface’. The evaluation of the ratio of ‘particle area’ to particle weight’ compares the peak area of the selected trace organic chemicals with the weighed-in particle mass. This allows conclusions to be drawn about the sorption capacity of the individual polymer types and sizes. The ratio peak area to particle surface reflects the peak areas of the trace organic chemicals on the calculated particle surface.

## 3. Results and Discussion

The aim of this study was to develop a method for the simultaneous analysis of trace organic chemicals on micro- and nanoplastic particles and polymer identification using a new analytical technique combining TD-GC/MS and Pyr-GC/MS. First, it had to be ensured that the method was reproducible and free of carry-over for the analysis of reference polymers. In a next step, this method was validated using selected trace organic chemicals that were sorbed onto reference particles. As indicator chemicals, in this study, we used phenanthrene, α-cypermethrin, and triclosan. The three trace organic chemicals were selected considering their different structural formulas, hydrophobicities (represented by different log K_OW_ values) and environmental relevance (Table 2). In this study, PS (41, 40, 0.078 µm), PE (48 µm) and PMMA (48 µm) particles were used.

### 3.1. Scope of Application for TD-Pyr-GC/MS

The application area of TD-Pyr-GC/MS is mainly in the rapid qualitative analysis of environmental samples targeting interactions of trace organic chemicals and different polymer types, e.g., ecotoxicological assays or spiked samples of lab- or pilot-scale wastewater treatment processes. Since no additional extraction steps are required for the analysis of trace organic chemicals, the sample preparation time is short. The pure measuring time for one sample is 2 h, which allows a high sample throughput. Figure 3 illustrates a possible workflow of sample analysis. The particles to be analyzed are separated from the liquid phase by filtration and freeze-drying. For analysis, the samples are applied to the pyrolysis tubes. Depending on the assay, a target analysis (SIM mode) or a non-target analysis (SCAN mode) can be performed. Then, the coupled TD-Pyr-GC/MS analysis is carried out as described in 2.1. The final data analysis results in a quick qualitative overview of possible trace organic chemicals present and the types of polymers.

### 3.2. Sources of Contamination in the TD-Pyr-GC/MS System

One aim of this study was to establish an analytical approach by minimizing carry-over. Preliminary experiments were carried out with PS containing styrene dimers and trimers as characteristic substances for identification in the MS fragment spectrum. An accumulation of the characteristic substances in the system was observed with increasing numbers of measurements. This carry-over was verified by measuring an empty tube with TD-GC/MS followed by Pyr-GC/MS after each measurement. The results imply that the analytes are transferred from incomplete pyrolysis of the sample to the subsequent TD measurement of the empty tube. It is assumed that during pyrolysis, not the whole content of substances reaches the GC column but accumulates in the system [43].

(a) Sample Inlet Tubes

Carryover was observed using tube types A and B (Figure 2b). Measurements with tube type C showed good results and significantly reduced the problem of substance transfer. Therefore, type C tubes were used for all temperature optimization tests and sorption experiments. Pyrolysis tubes type A and B are closed at the bottom. Therefore, a constant flow of helium cannot be guaranteed.

(b) Transport Adapters

Pyrolysis tubes type C were mounted on transport adapters (Figure 2c) which can also be a source of contamination. To avoid contamination inside of the adapter, the lower part of the adapter was cleaned after each measurement for 15 min in dichloromethane and an ultrasonic bath. In addition, the filament of the pyrolysis unit is another wearing part carrying a risk of contamination.

(c) Filament for pyrolysis

After approximately 300–350 measurements with the same filament, more impurities appeared during the empty tube measurements. The filament was examined more closely and black dots and soot discolorations were visible. However, this source of contamination could easily be eliminated by changing the filament regularly.

### 3.3. Influence of Different TD Temperature Programs on Pyrolysis of Particles of Different Materials and Size

**Figure 4:**
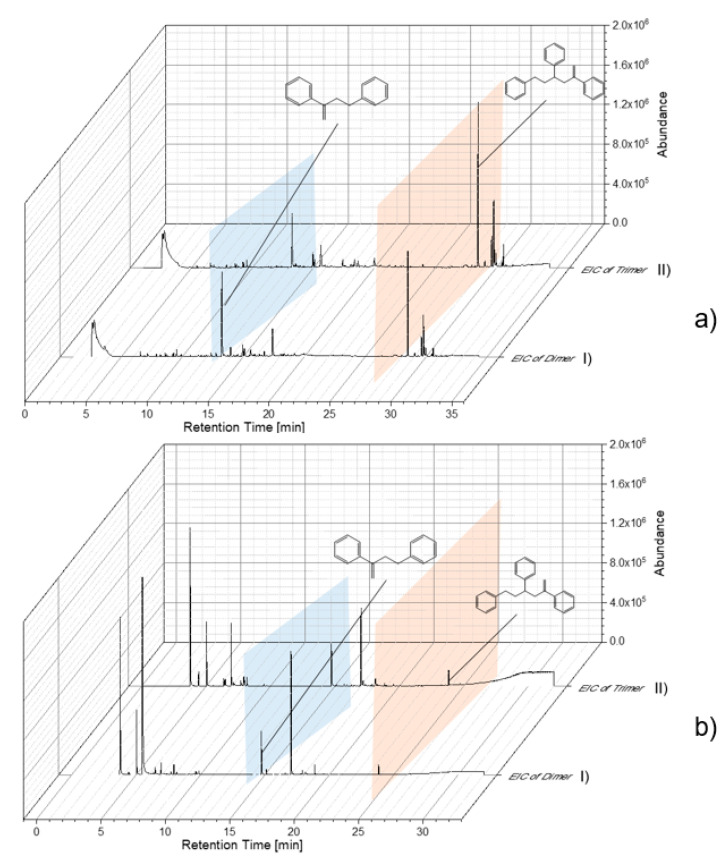
Evaluation of chromatogram/pyrogram of PS 78-nm particles by extracted-ion chromatogram (EIC) of dimer (I) and Trimer (II) (**a**) TD-Chromatogram (TD-GC/MS), (**b**) Pyrogram (Pyr-GC/MS).

Reference particles without indicator substances were used to study and optimize the thermal desorption temperature. Thereby, characteristic pyrolysis products of each polymer were used for the Pyr-GC/MS data evaluation. Particle samples with adsorbed substances were thermodesorbed (TD-GC/MS) and subsequently pyrolyzed (Pyr-GC/MS). In this way, already, pyrolysis fragments eluting in the TD-GC/MS could be identified. The resulting chromatograms and pyrograms of the individual polymers were extracted according to their characteristic fragments (as shown in Table 1). As examples, PS chromatograms and pyrograms are shown in Figure 4. These were extracted for the di- and trimer in TD (Figure 4a) and Pyr (Figure 4b), respectively. The pyrogram shown in Figure 4a reveals that polystyrene particles (78 nm) are, in large part, already fragmented into the di- and trimer in the TD. In Figure 4b, the pyrogram is contrasted with the di- and trimer of the polystyrene particle (78 nm).

**Figure 5:**
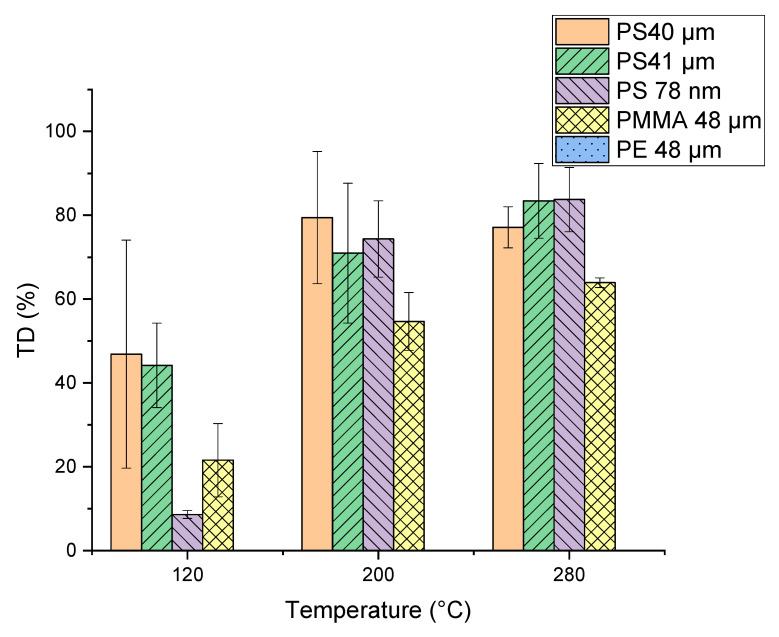
Percentage of the characteristic pyrolysis products of the polymers (Table 1) of the single polymers (polymethyl methacrylate (PMMA), polyethylene (PE) and polystyrene (PS)) already visible in the TD Chromatogram calculated from the peak areas. The remaining percentage of the characteristic pyrolysis products is visible in the Pyrogram.

For TD temperature optimization, measurements with the selected particles (PMMA, PE and PS) were performed at the final temperatures of 120, 200 and 280 °C and results are shown in Figure 5. A significant increase in PMMA pyrolysis products in the thermodesorption step occurred with an increase in TD temperature. Differences can be seen for the PS microplastic particles (PS 40 µm and PS 41 µm). Since the PS 40 µm particles were stored dry but the PS 41 µm were suspended in ethanol, it seems that the storage conditions of the particles might have an influence. The TD temperature variation has no influence on the PE particles, independent of the temperature the characteristic products are only visible in the pyrogram. The final thermal desorption temperature was chosen at 200 °C, considering that 120 °C is too low to achieve quantitative desorption of most trace organic chemicals. A TD temperature of 200 °C is also more suitable than 280 °C for the analysis of thermolabile substances.

### 3.4. Desorption Behavior of Phenanthrene, α-Cypermethrin and Triclosan

**Figure 6:**
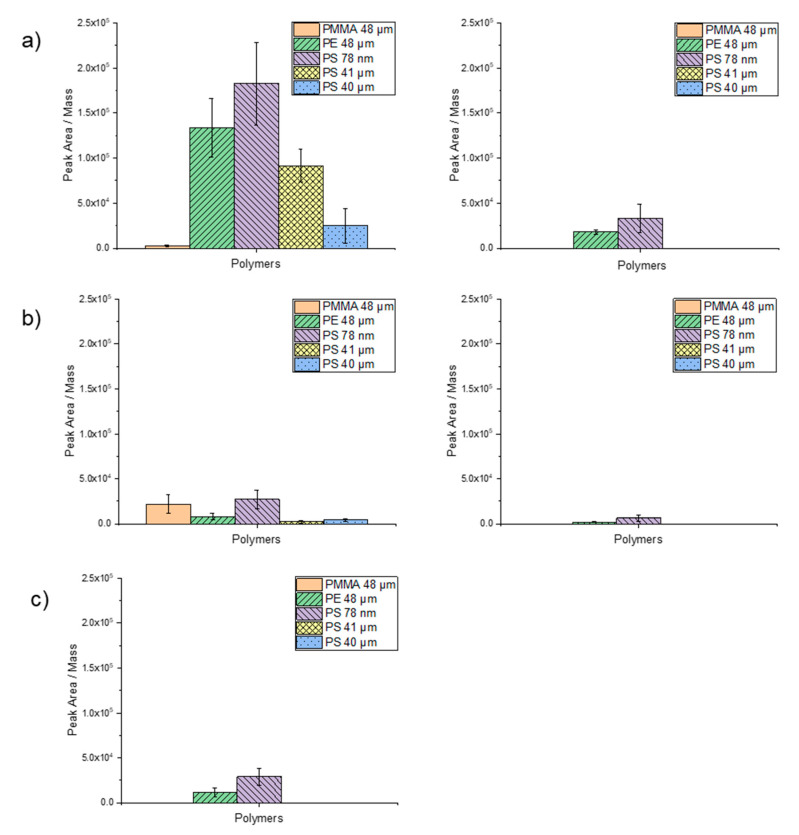
Sorption of the selected trace substances on PMMA (48 µm), PE (48 µm), PS (78 µm), PS (41 µm) and PS (40 µm) particles: (**a**) 1 µg/mL (left) and 0.1 µg/mL (right) phenanthrene, (**b**) 1 µg/mL (left) and 0.1 µg/mL (right) α-cypermethrin and (**c**) 1 µg/mL triclosan. For evaluation, the peak area of the trace substance was divided by the weighed mass.

For the validation of the newly developed TD-Pyr-GC/MS method, the sorption behavior of the target trace organic chemicals (Table 2) at two final concentrations (1000 µg/L and 100 µg/L) on the different particles was investigated. The boiling points of the trace organic chemicals used in this study are reported in Table 2. If more polymer products are present in the TD, there is a risk of interference with trace organic chemicals and that both the trace organic chemicals and the characteristic substances of the polymers can no longer be clearly identified. Figure 6a–c illustrate the measured peak area of the trace organic chemicals divided by the weighed mass for the three target trace organic chemicals. The results are discussed in the following sections.

Phenanthrene at a concentration of 1000 µg/L exhibited the highest degree of peak area per mass on PS nanoparticles (78 nm), followed by PE (48 µm), PS (41 µm) and PS (40 µm). Almost no sorption occurred on PMMA (48 µm) (Figure 6a). These findings suggest a significant influence of particle size on sorption ability. For phenanthrene at a concentration of 100 µg/L, only sorption on PS 78 nm and PE particles was observed. The lowest degree of sorption of phenanthrene was noticed on the PMMA particles. A difference was also observed for the PS particles, although they almost have the same size (i.e., 40 and 41 µm). The particles suspended in ethanol (PS 41 µm) exhibited a higher degree of sorption than the dry particles (PS 40 µm). Here, ethanol might have served as an adsorption mediator.

The normalized degree of sorption of 1000 µg/L α-cypermethrin on the particles was in general lower compared to phenanthrene (Figure 6b). Applying α-cypermethrin at a concentration of 100 µg/L sorption was only observed on PS 78 nm and PE particles. Due to a more complex structure and the higher molecular weight of α-cypermethrin compared to phenanthrene, a contact time of 1 h may have been too short to achieve a higher degree of adsorption. Interestingly, however, sorption is higher on PMMA and lower on PE. Here, too, the highest degree of sorption was observed on PS 78-nm particles.

Triclosan at a concentration of 1000 µg/L adsorbed only on PS 78-nm and PE 48-µm particles within a contact time of 1 h (Figure 6c). Applying triclosan at a concentration of 100 µg/L, no sorption was observed on any particle type. This contact time was likely too short, since Li et al. (2019) reported that the sorption equilibrium of triclosan was only reached after 72 h [44]. Nevertheless, also for triclosan, the highest degree of sorption was observed for the nanoparticles.

Due to the relatively short contact time of 1 h, the sorption equilibrium may not yet have been reached and further experiments with longer contact times are pending. However, the sorption kinetics and the individual detection limits of trace substances are beyond the scope of this paper and are currently under investigation.

### 3.5. Comparison of the Desorption Behavior of the Selected Trace Organic Chemicals in TD and PYR

**Figure 7:**
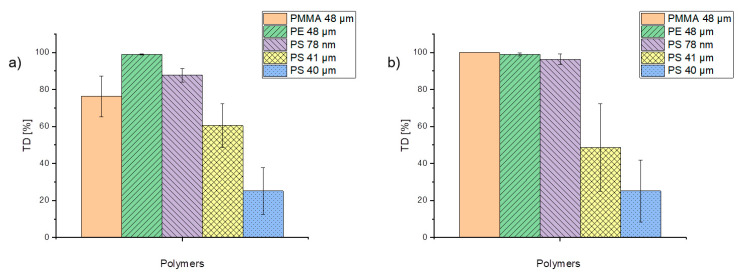
Percentage of phenanthrene in the concentrations of 1 µg/mL (**a**) and 0.1 µg/mL (**b**) desorbed during TD from the particles.

In order to assess the tendency of trace organic chemicals to desorb from particles, the percentage of phenanthrene desorption during TD was considered and is illustrated in Figure 7. Although values fluctuate, almost 100% of phenanthrene was already desorbing from PE particles during the TD step, while PS particles (41 and 40 µm) exhibited desorption varying between 25 and 60%. These significant differences may be due to the different pretreatments of the particles. The PS 41-µm particles were suspended in ethanol whereas the PS 40-µm particles were stored dry. By comparing different concentration levels of applied trace organic chemicals, the concentration does not seem to influence the desorption for PE, PS 40-µm, and PS 78-nm particles significantly (Figure 7). These findings suggest that the desorption behavior depends on both the particle size and the particle type. α-cypermethrin and triclosan were both completely desorbed within the TD (data not shown).

On the basis of these experiments, it is not yet possible to draw any conclusions regarding the quantity of trace organic chemicals adsorbed on microparticles. However, those conclusions will potentially be possible in the near future when both the trace organic chemicals on the particles and the remaining trace organic chemicals in the aqueous phase will be examined. The aqueous phase is analyzed via stir bar sorptive extraction (SBSE) and TD-GC/MS, the particles via TD-Pyr-GC/MS. It was demonstrated that desorption behavior depends on both the particle type and the particle size. In addition to size and shape, the main characteristics that are considered to influence sorption on a plastic particle are crystallinity, density, structure and hydrophobicity [5,10,45].

### 3.6. Comparison of SCAN and SIM Mode for Selected Trace Organic Chemicals on PS Nanoparticles

The three target trace organic chemicals were incubated at the concentration of 1000 µg/L on PS 78 nm particles and measured in TD-GC/MS using SIM mode. The SIM mode was optimized for the respective trace substance; see Section 2.4. The peak areas, and thus the sensitivity, increased in SIM mode for all trace substances: phenanthrene (+33% ± 2%), α-cypermethrin (+54% ± 12%), and triclosan (+58% ± 12%). For target analysis, it is, therefore, recommended to measure in SIM mode.

### 3.7. Trace Organic Chemical Sorption in Relation to the Particle Surface

Since the polystyrene particles are assumed to be spherical in shape, their surface area could be calculated. Subsequently, the calculated surface was divided by the measured peak area in the chromatogram. The number of particles was calculated on the basis of the weighed mass (Table 3). As expected, the number of nanoparticles (78 nm) is significantly higher compared to microparticles (40 and 41 µm). In addition, the calculated surface area of the nanoparticles confirms that the surface area is significantly larger for almost the same mass, thus providing more opportunities for the trace organic chemicals to adsorb.

**Figure 8:**
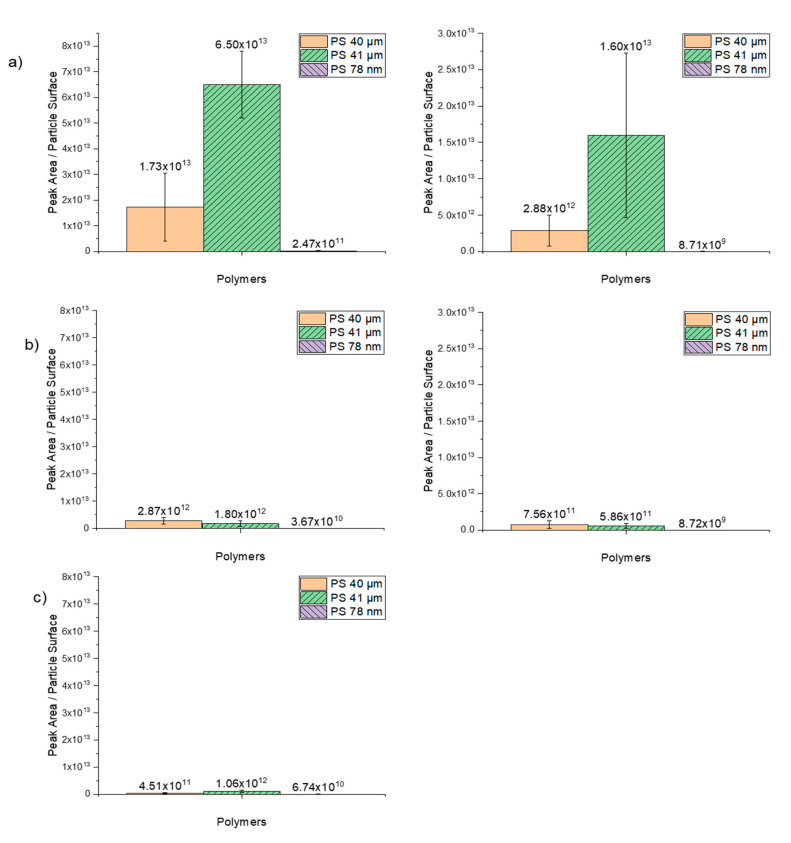
Sorption of the selected trace substances as a function of peak area/particle surface on the spherical PS particles in sizes 40 µm, 41 µm and 78 nm. (**a**) 1 µg/mL (right) and 0.1 µg/mL (left) phenanthrene, (**b**) 1 µg/mL (right) and 0.1 µg/mL (left) α-cypermethrin, (**c**) 1 µg/mL (right) triclosan.

The degree of sorption of trace organic chemicals as a function of the peak area per particle surface is illustrated in Figure 8. Comparing these findings with the results in which the polymers were plotted as a function of particle per mass reveals that the higher degree of sorption of nanoparticles is mainly due to the high number of particles and the higher specific surface area. The results also suggest that the sorption on PS 41-µm particles suspended in ethanol works better than on dry 40-µm particles.

## 4. Conclusions and Outlook

The results of this study confirm that a new method can simultaneously identify trace organic chemicals and polymer type in one analytical setup. This method offers fast sample analysis within a short time period of 2 h. In comparison to Herrara et al., 2003 and Fries et al., 2013, who use a double shot pyrolysis or a sequential pyrolysis GC/MS for the characterization of additives in polymers, the sorption behavior of trace organic substances on different polymers and sizes without extraction steps could be shown for the first time in this work [12,14]. The method development for the determination of the optimal TD-temperature for the TD-Pyr-GC/MS also showed that this temperature significantly influences the desorption behavior. In addition, it could be shown that TD-Pyr-GC/MS is particularly suitable for the analysis of nanoplastic particles. The following objectives were achieved through the TD-Pyr-GC/MS method development:

1. Temperature optimization of the proposed TD-GC/MS method requires that the thermal desorption temperature should be as high as possible in order to desorb all sorbed substances, and at the same time, the temperature must be so low that as few pyrolysis products as possible are generated during the thermal desorption step. In this regard, the optimal TD-temperature was identified to be 200 °C.
2. A suitable Pyr-GC/MS method was developed which completely depolymerizes all targeted polymers (i.e., PS, PE and PMMA) without leaving residues in the system and therefore avoiding carry-over issues. For this purpose, an optimum pyrolysis temperature of 800 °C was determined.

In the experiments carried out, it was confirmed that sorption of trace organic chemicals depends on both particle size and polymer type. In all sorption tests, it could be shown that the sorption on nanoplastic particles was highest. Phenanthrene and α-cypermethrin exhibited a higher degree of sorption on PS nanoparticles and on PS microparticles. For triclosan, no sorption on PMMA and PS microplastic particles was observed. The sorption of phenanthrene on the particles was strongly polymer-dependent (PMMA << PS 40 µm < 41 µm < PE < PS 78 nm). This adsorption trend was also observed for α-cypermethrin (PS 41 µm < PS 40 µm < PE < PMMA < PS 78 nm). Triclosan adsorbed only on PE and PS 78-nm nanoplastic particles (PE < PS78). In this study, however, the contact time was limited to one hour and the sorption equilibrium might not have been reached after this time period. The focus of this study, however, was not on sorption kinetics but on the establishment and practical application of a new method for the analysis of trace organic chemicals on micro- and nanoplastic particles. In further experiments, the contact time should be extended in order to assure that the sorption equilibrium between the trace organic chemicals on the particles and the aqueous phase is reached and to elucidate sorption kinetics.

## Figures and Tables

**Table 1:** Characteristic pyrolysis products of selected polymers for identification.

Polymer Type	Characteristic Pyrolysis Fragments	Formula	Molecular Weight (g/mol)	*m/z* (Intensity Ratio (%)) *	Structure
PS	3-butene-1,3-diyldibenzene (styrene dimer)	C_16_H_16_	208	91 (100), 104 (27), 130 (23), 208 (30)	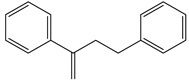
PS	5-hexene-1,3,5-triyltribenzene (styrene trimer)	C24H24	312	91 (100), 117 (32), 194 (19), 207 (25)	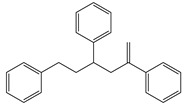
PE	1,12-tridecadiene	C13H24	180	55 (52), 81 (44), 67 (38), 95 (26)	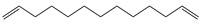
PE	1,13-tetradecadiene	C14H26	194	81 (42), 95 (27), 109 (13)	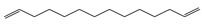
PE	1,15-hexadecadiene	C16H30	222	55 (63), 81 (50), 96 (45), 69 (37)	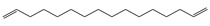
PMMA	Methyl methacrylate	C_5_H_8_O_2_	100	41(77), 69 (100), 100 (57)	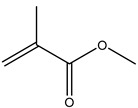

* intensity ratio to largest peak in spectra.

**Table 2:** Characteristic signals, properties and structure of chosen substances.

Substance	Characteristic Signals (*m/z*)	Molecular Weight (g/mol)	Log D Value (pH 5.5) *	Environmental Relevance	Boiling Point (°C) at 760 mmHg	Structure
Phenan-threne	178	178	5.27	High toxicity, mutagenic [39], typical waste water pollutant [9]	337.4 ± 9.0	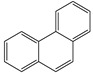
α-Cyper-methrin	163, 184, 209	416	6.05	The most widespread product of Type II pyrethroid pesticide [9,40]	511.3 ± 50	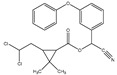
Triclosan	290, 288, 218, 63	290	5.27	Antimicrobial agent which is used in personal care products [41,42]	344.6 ± 42.0	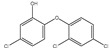

* values from chemspider.com database.

**Table 3:** Mass and number of polystyrene particles.

Particle Size (µm)	Particle Type	Mass (µg)	Number of Particles	Surface Particles (m^2^)
41	PS	22–63	586–1679	3.10 × 10^−6^–8.86 × 10^−6^
40	PS	23–64	660–1837	3.32 × 10^−6^–9.23 × 10^−6^
0.078	PS	29–69	1.12 × 10^11^–2.67 × 10^11^	2.14 × 10^−3^–5.10 × 10^−3^
48	PE	Not spherical		
48	PMMA	Not spherical		
